# Trend of leprosy detection rate in Brazil, 1990 to 2016^☆^^☆☆^

**DOI:** 10.1016/j.abd.2018.10.003

**Published:** 2019-09-30

**Authors:** Carlos Dornels Freire de Souza, Franklin Gerônimo Bispo Santos, Thiago Cavalcanti Leal, João Paulo Silva de Paiva

**Affiliations:** a Department of Medicine, Universidade Federal de Alagoas, Arapiraca, AL, Brazil; b Universidade Federal de Alagoas, Arapiraca, AL, Brazil

**Keywords:** Epidemiology, Leprosy, *Mycobacterium leprae*

## Abstract

The objective of this study was to describe the trend of detection of the disease in Brazil from 1990 to 2016. The joinpoint regression model was used. There was a significant trend of decreased detection in the country (average annual percent change  −1.8%) and in the South (average annual percent change = −3.5%) and Southeast regions (average annual percent change = −4.5%). The Northeast (average annual percent change = 0.2%), the Central-West (average annual percent change = −1.5%), and the North (average annual percent change = −2.6%) showed a stationary trend (*p* > 0.05). Eleven states showed a decreasing trend. Alagoas (average annual percent change = 2.1%) and Rio Grande do Norte (average annual percent change = 1.4%) presented significant increase (*p* < 0.001). The heterogeneous pattern of trend between regions and states shows that efforts are needed to eliminate the disease.

Leprosy is a neglected tropical disease that represents a public health problem in several developing countries. Brazil occupies an undesirable position in the global scenario, being one of the 13 countries that comprised 94% of all new cases registered in the world in 2014.^1^ In 2016 alone, 25,218 new patients were reported, a detection rate of 12.23/100,000 inhabitants.^2^

The leprosy detection rate reflects the magnitude of the disease in the territory. Its relevance is recognized by the World Health Organization (WHO) as an instrument that enables the systematic monitoring of the efficiency of control programs, as well the fulfillment of the established goals. In this sense, the study of the trend of detection rates is in line with the first pillar of the global initiative that aims strengthen control, coordination, and partnership with government.^1^, ^2^

Therefore, this work aimed to describe the temporal trends of the detection rates of new cases of leprosy in the general population in Brazil, and its regions and states, from 1990 to 2016.

The rates of detection of new cases of leprosy were analyzed in the general population: national, regional, and state. For the analysis, a segmented regression model was adopted.^3^, ^4^ The annual percentage change (APC) and the average annual percent change (AAPC) were calculated with a 95% confidence interval (95% CI) and 5% significance. The trend was classified as increasing, stationary, or decreasing. In addition, the average rate of the period (ratio between the sum of the annual rates and the total number of years in the period studied) was calculated.

There was a statistically significant reduction in the detection rate at the national level (AAPC = −1.8%; *p* < 0.001), decreasing from 19.96 to 12.23 new cases per 100,000 inhabitants (Fig. 1). The decrease has been observed not only in Brazil, but also throughout the world, as a result of the commitment of the WHO, materialized in strategies and actions directed toward disease control, especially multidrug therapy, recommended by the WHO in the early 1980s and implemented in Brazil in 1991.^1^, ^5^ Although the advances are a reality, the average detection rate of the period (1990–2016) was 22.13/100,000 inhabitants, classifying the country as having very high endemicity (Fig. 2 and Table 1).

**Figure 1:**
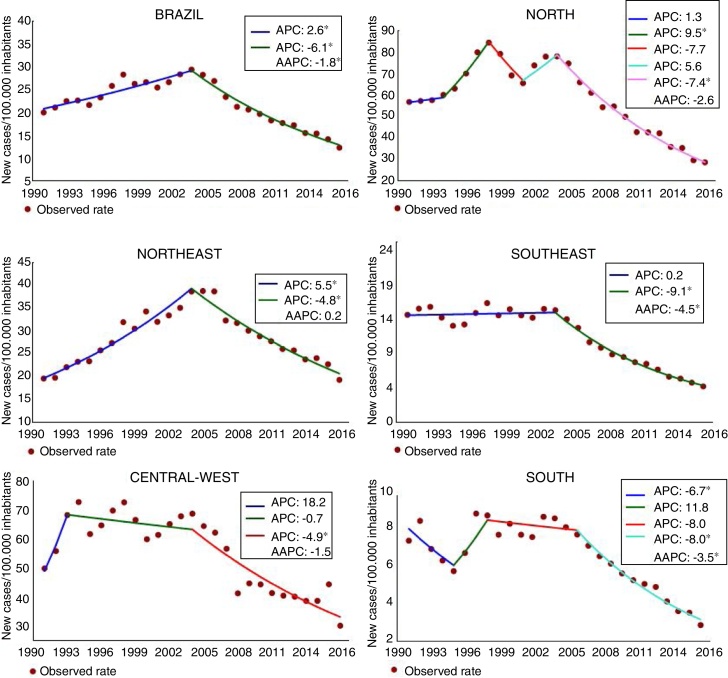
Trend of detection rate of new leprosy cases in the general population per 100,000 inhabitants. Brazil, 1990–2016. APC, annual percent change; AAPC, average annual percent change. *Statistically significant.

**Figure 2:**
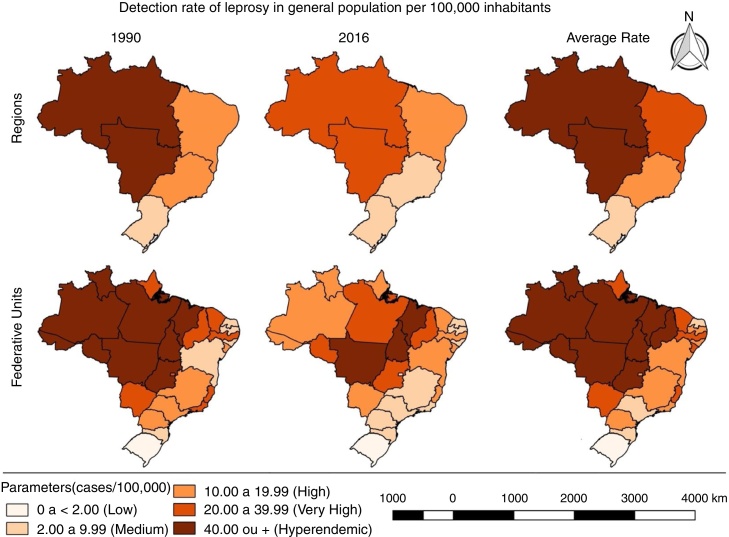
Spatial pattern of detection rates of new leprosy cases in the general population of Brazil, from 1990 to 2016.

**Table 1:** Trend of detection rate of new cases of leprosy/100,000 inhabitants in Brazil, 1990–2016.

Brazil/Regions/States	Rate per 100,000 inhabitants			AAPC	95% CI	Trend
	1990	2016	Average			
*Brazil*	19.96	12.23	22.13	−1.8^a^	−2.3 to −1.4	Decreasing

*North*	56.81	28.70	58.92	−2.6	−4.6 to 0.5	Stationary
Rondônia	53.38	26.63	75.71	−2.0^a^	−3.9 to 0.0	Decreasing
Acre	87.63	14.20	54.30	−6.7^a^	−7.9 to −5.5	Decreasing
Amazonas	76.33	11.20	40.11	−6.9^a^	−7.8 to −5.9	Decreasing
Roraima	49.52	16.34	54.19	−4.7^a^	−7.8 to −1.4	Decreasing
Pará	45.48	30.43	61.92	−1.3	−4.8 to 2.4	Stationary
Amapá	33.43	11.50	31.57	−3.7	−11.6 to 4.8	Stationary
Tocantins	72.06	88.13	85.05	0.5	−3.4 to 4.6	Stationary

*Northeast*	19.60	19.30	28.40	0.2	−0.3 to 0.7	Stationary
Maranhão	44.29	47.43	67.15	0.4	−0.5 to 1.2	Stationary
Piauí	32.18	27.64	45.78	−0.6	−1.6 to 0.5	Stationary
Ceará	24.10	18.94	28.42	−0.9^a^	−1.5 to −0.2	Decreasing
Rio Grande do Norte	4.40	5.70	8.23	1.4^a^	0.1 to 2.7	Increasing
Paraíba	9.56	9.63	17.99	0.6	−1.7 to 2.8	Stationary
Pernambuco	26.20	19.72	33.56	−0.2	−1.5 to 1.0	Stationary
Alagoas	5.89	8.13	10.62	2.1^a^	0.1 to 4.2	Increasing
Sergipe	11.46	13.73	22.48	1.0	−1.6 to 3.6	Stationary
Bahia	9.92	13.60	16.46	1.7	−0.6 to 4.1	Stationary

*Central-West*	49.79	30.02	55.22	−1.5	−3.0 to 0.9	Stationary
Mato Grosso do Sul	28.37	15.21	27.70	0.3	−2.3 to 3.0	Stationary
Mato Grosso	101.65	80.62	113.33	−0.5	−3.0 to 2.0	Stationary
Goiás	44.34	21.69	54.07	−2.3	−5.3 to 0.7	Stationary
Distrito Federal	21.80	5.91	13.96	−5.1^a^	−5.8 to −4.3	Decreasing

*Southeast*	14.47	4.17	11.50	−4.5^a^	−5.1 to −4.0	Decreasing
Minas Gerais	12.42	5.34	13.00	−4.3^a^	−5.1 to −3.4	Decreasing
Espírito Santo	36.40	10.97	33.54	0.6	−9.5 to 11.8	Stationary
Rio de Janeiro	21.88	4.33	17.99	−6.3^a^	−9.5 to −3.0	Decreasing
São Paulo	10.67	3.95	6.63	−4.8^a^	−5.4 to −4.1	Decreasing

*South*	7.25	2.84	6.49	−3.5^a^	−5.7 to −1.2	Decreasing
Paraná	3.43	5.20	12.95	−3.6^a^	−6.4 to −0.8	Decreasing
Santa Catarina	6.63	2.13	3.90	−4.1^a^	−7.3 to −0.7	Decreasing
Rio Grande do Sul	1.82	0.92	1.77	−2.3^a^	−3.2 to −1.5	Decreasing

AAPC, average annual percent change.

a Statistically significant.

In the regional analysis, the North had the highest average rate (58.92/100,000). followed by the Central-West (55.22/100,000). In turn, the South presented the lowest rate (6.49/100,000). In the last year of the series, in the regions of North, Northeast, and Central-West, the endemic was classified as very severe. In addition, the Southeast and South registered average endemicity. In the analysis by the joinpoint, reduction tendencies were observed in the South (AAPC = −3.5%; *p* < 0.001) and in the Southeast (AAPC = −4.5%; *p* < 0.001; Figure 1, Figure 2; Table 1).

Considering the Brazilian states, the highest average rate was observed in Tocantins (85.05/100,000), increasing from 72.06/100,000 in 1990 to 88.13/100,000 in 2016. In seven other states, the 2016 rates were higher than those observed at the beginning of the period studied: Maranhão, Rio Grande do Norte, Paraíba, Alagoas, Sergipe, Bahia, and Paraná. In parallel, in the last year of the series, together with Tocantins, hyperendemic conditions were recorded in Mato Grosso (80.62/100,000) and Maranhão (47.43/100,000; Fig. 2 and Table 1).

There were verified reduction trends in 11 states and the Federal District: four in the North, one in the Northeast, three in the Southeast, three in the South, and the Federal District in the Central-West. Amazonas presented the largest reduction (AAPC = −6.9%; *p* < 0.001), decreasing from 76.33/100,000 in 1990 to 11.12/100,000 in 2016, for an average rate of 40.11/100,000 (Fig. 2 and Table 1).

Alagoas and Rio Grande do Norte presented statistically significant growth trends. In Alagoas, the rate increased from 5.89/100,000 in 1990 to 8.13/100,000 in 2016 (AAPC = 2.1%; *p* < 0.001), and in Rio Grande do Norte it increased from 4.40/100,000 in 1990 to 5.7/100,000 in 2016 (AAPC = 1.4%; *p* < 0.001). It is worth noting that in these two states, leprosy has already reached the level of elimination (prevalence < 1 case/10,000 inhabitants; Fig. 2, Table 1). This finding indicates that the chain of transmission in these states is active, suggesting operational problems and pointing to a hidden prevalence.

The asymmetries observed in the detection of the disease in Brazil signal to the complexity of the problem. Recently, researchers have alerted of the hidden high prevalence of the disease and underdiagnosis in the country.^6^, ^7^ The increasing trends observed in Alagoas and Rio Grande do Norte and the stationary pattern in 13 federative units and in North, Northeast, and Central-West regions signal the need for interventions in these areas in order to interrupt the transmission chain.

## Financial Support

None declared.

## Author's contribution

Carlos Dornels Freire de Souza: Statistical analysis; approval of the final version of the manuscript; conception and planning of the study; elaboration and writing of the manuscript; obtaining, analyzing and interpreting the data; effective participation in research orientation; intellectual participation in propaedeutic and/or therapeutic conduct of the cases studied; critical review of the literature; critical review of the manuscript.

Franklin Gerônimo Bispo Santos: Approval of the final version of the manuscript; conception and planning of the study; elaboration and writing of the manuscript; critical review of the literature; critical review of the manuscript.

Thiago Cavalcanti Leal: Statistical analysis; approval of the final version of the manuscript; conception and planning of the study; elaboration and writing of the manuscript; obtaining, analyzing and interpreting the data; effective participation in research orientation; intellectual participation in propaedeutic and/or therapeutic conduct of the cases studied; critical review of the literature; critical review of the manuscript.

João Paulo Silva de Paiva: Statistical analysis; approval of the final version of the manuscript; conception and planning of the study; elaboration and writing of the manuscript; obtaining, analyzing and interpreting the data; effective participation in research orientation; intellectual participation in propaedeutic and/or therapeutic conduct of the cases studied; critical review of the literature; critical review of the manuscript.

## Conflicts of interest

None declared.
